# Transcriptional profiling of biofilms formed on chilled beef by psychrotrophic meat spoilage bacterium, *Pseudomonas fragi* 1793

**DOI:** 10.1016/j.bioflm.2021.100045

**Published:** 2021-02-17

**Authors:** Nirmani N. Wickramasinghe, Joshua Ravensdale, Ranil Coorey, Gary A. Dykes, Peter S. Chandry

**Affiliations:** a School of Public Health, Curtin University, Bentley, 6102, Western Australia, Australia; b CSIRO, Agriculture and Food, Werribee, 3030, Victoria, Australia; c School of Molecular Sciences, Curtin University, Bentley, 6102, Western Australia, Australia; d Graduate Research School, Curtin University, Bentley, 6102, Western Australia, Australia

**Keywords:** Meat, Biofilms, *Pseudomonas fragi*, RNA sequencing, Differential gene expression

## Abstract

*Pseudomonas fragi* is the predominant bacterial species associated with spoiled aerobically stored chilled meat worldwide. It readily forms biofilms on meat under refrigerated temperature conditions used in the meat industry. Biofilm growth leads to slime development on meat which in turn becomes a major quality defect. To understand the genetic regulation that aids *P. fragi* to survive under chilled conditions used in the meat industry, as well to obtain an overview of the transcriptomic behavior of this organism when grown as biofilms, RNA sequencing was carried out for the main stages of the *P. fragi* 1793 biofilm. RNA was extracted at different stages of the biofilm cycle namely initiation, maturation and dispersal. At the same time, the biofilm growth was assessed by fluorescent staining and imaging using confocal laser scanning microscope. The results of RNA sequencing were verified by qRT-PCR using twelve genes that were most significantly up and down regulated at each stage. Differential expression analysis at biofilm maturation revealed 332 significantly upregulated genes and 37 downregulated genes relative to initiation. Differential expression analysis at biofilm dispersal reveled 658 upregulated and 275 downregulated genes relative to initiation. During biofilm maturation and dispersal, genes coding for flp family type IVb pilin, ribosome modulation factor, creatininase were the most upregulated genes while genes encoding for iron uptake systems including TonB-dependent siderophore receptor and taurine transport were significantly down regulated. The results show that protein synthesis and cellular multiplication cease after the biofilm population maximum has reached.

## 1 Introduction

Psychrotrophic *Pseudomonas* species are the key spoilage organisms in aerobically stored chilled meat [1]. These species are equipped with many metabolic traits which aid them in withstanding the harsh environmental conditions of low temperature storage and in overcoming competition from other psychrotrophic spoilage organisms [2]. Among the psychrotrophic spoilage *Pseudomonas* species, *Pseudomonas fragi* is the most frequently encountered spoilage species on meat including beef, chicken, pork, lamb and fish, worldwide [3]. *Pseudomonas fragi* readily forms biofilms on meat under refrigerated temperature conditions used in the meat industry [4]. When biofilms combine with meat exudate, it results in slime formation which is a key quality defect that leads to consumer rejection of meat.

Biofilms are formed when planktonic bacteria attach to a surface or to each other and embed themselves in a self-produced or an acquired exo-polymeric matrix [5]. Biofilms protect the residing bacteria from harsh environmental conditions including desiccation, radiation, predation and antimicrobial compounds [6]. It is likely that biofilm formation aids *P. fragi* to survive stressful environmental conditions and become the predominant spoilage flora on long-term stored chilled meat.

When studying the characteristics of bacterial biofilms, it is important to study them *in situ* or using industry applicable experimental model systems. The metabolic reactions, rates and by-products as well as expression of genes can vary greatly based on the environmental conditions that they are grown under [7]. Therefore, it is important to design model systems that closely mimic the practical spoilage conditions in the meat industry.

Biofilms undergo key distinct stages in their life which include irreversible attachment, initiation, maturation and dispersal [8]. Each of these stages are controlled by specific set of genes which regulates different metabolic functions at each of these stages. Currently, limited information is available about the genetic regulation of *P. fragi* biofilm formation under industry applicable conditions. A main objective of this study was to identify gene expressions that may aid the survival of *P. fragi* biofilms on meat kept under low temperature conditions. Furthermore, recent studies have found that despite having continues access to nutrients biofilms formed on meat by psychrotrophic *P. fragi* dispersed after reaching a population maximum [4].

Furthermore, to comprehend the molecular mechanisms involved in these transitions between biofilm to dispersed stage as well as to come up with potential inhibitory compounds that can be used to limit biofilm formation on meat, the present study examines the global gene expression of the biofilm cycle of *P. fragi* strain 1793, when formed on raw beef aerobically stored at temperature abuse conditions. This was achieved by pairwise comparisons of the transcriptomes of key stages of the biofilms cycle which include initiation, maturation and dispersal. Genes that were differentially expressed at each stage were further evaluated for functional classes using the National Center for Biotechnology (NCBI) Cluster of Orthologues Groups of Proteins (COGS) database. An increased understanding of *P. fragi* biofilm formation and dispersal under low temperature conditions can help to design potential biofilm inhibitory compounds that can be used to limit slime formation on meat. To the best of our knowledge, the data presented in this study provide the first report of the gene expression profile of *P. fragi* biofilms formed on chilled beef.

## 2 Material and methods

### 2.1 Biofilm growth on meat

Based on our previous work, *P. fragi* strain 1793 was selected to study RNA sequencing due to its rapid and dense biofilm formation on meat [4]. Beef ‘eye round’ cut was used throughout the series of experiments to minimize the variability that can arise from the composition of different cuts. The beef was purchased from local butchers as 3 ​kg cuts and brought chilled to the laboratory within 20 ​min. The meat was kept at 3 ​°C until processing. Since the interior tissues of healthy living animals are considered free from microorganisms (Gill & Penney, 1977; Mackey & Derrick, 1979), a surface sterilization method was applied. The beef was surface sterilized by immersing the meat in boiling water for 10 ​min. Meat was removed from water, the cooked exterior was aseptically excised and was sliced into 3 ​mm thick cuts using a sterilized, stainless steel deli-slicer placed inside a laminar flow hood. The meat slices were further sectioned into circular shapes with a diameter of 10 ​cm and were placed in petri plates.

Overnight cultures of *P. fragi* 1793 were prepared by inoculating a single colony into 5 ​ml of tryptone soy broth (TSB, Oxoid, Basingstoke, United Kingdom) and incubating it at 25 ​°C for 18 ​h in a shaking incubator at 180 ​rpm. Then the cultures were decimally diluted, and each meat slice was inoculated with 1 ​ml of culture containing approximately 10^4^ ​CFU ​ml^−1^. Then the slice were spread plated covering the entire meat surface. The petri plates were covered with lids and meat slices were incubated at 10 ​°C in a static incubator. Biofilms were extracted from meat as described below at 48, 76 and 115 ​h, which correlate to time points of biofilm initiation, maturation and dispersal [4].

### 2.2 Biofilm staining and imaging

To assess the structural and cellular transformations over time of biofilms formed on meat, meat samples incubated at 10 ​°C were imaged from day one to day seven. At each time point, the samples were prepared for Confocal Laser Scanning Microscope (CLSM) imaging using a Leica SP5 (Leica Microsystems, Heidelberg, Germany) microscope. Live/Dead® BacLight™ Kit (L7012, Molecular Probes-Life Technologies, Eugene, OR, USA) was used to stain the biofilms. The stained samples were incubated in the dark at around 25 ​°C and placed on glass slides. A cover slip was gently placed on top of each muscle slice and samples were imaged under the 100x oil immersion objective.

Stained meat samples were excited with the 488 ​nm laser line from an Argon laser at 20% intensity. The samples were scanned at 200 ​Hz speed and imaged using 1200 ∗ 1200 resolutions. The emission wavelength was collected between 490 and 590 ​nm for SYTO 9 (displayed green for live cells) and between 600 and 650 ​nm for PI (displayed red for dead cells). Images (Z stacks) were acquired from six different locations on the meat surface which included the centre, edges and in between.

### 2.3 RNA extraction process

Samples for RNA sequencing were taken at biofilm initiation (48 ​h), maturation (76 ​h) and dispersal (115 ​h) stages. The time points of extraction were selected based on biofilm cell numbers calculated in previous cell cycle studies [4]. For each time point, four biological replicates were obtained in four different weeks which gave a total of 12 samples. RNA was extracted with the RNeasy minikit (Qiagen, MD, cat no-74104) using the protocol for disruption of bacteria grown on solid media, according to manufacturer’s instructions. A stabilization mixture of the RNA protect bacteria (Qiagen, MD) and TSB was added to the meat surface with the biofilm. Then the biofilm was gently extracted using a sterile cell scraper and pipetted into a centrifuge tube. The tube was vortex mixed and then centrifuged at 5000 ​g for 10 ​min.

Additional changes were made to the manufacturer’s protocol for enzymatic lysis and proteinase K digestion, which include the addition of 15 ​μl of Proteinase K (Qiagen, MD) and an increase in the amount of lysozyme to 15 ​mg ml^-^1 in the TE buffer. Then the biofilm was mechanically disrupted using a tissue lyser (SpeedMill) for 5 ​min ​at maximum speed. Genomic DNA was removed using on column DNase digestion with DNAse digestion kit (Qiagen, MD, cat no 79254). After extraction, total RNA yield was quantified using a Nanodrop (ND-1000, ThermoFisher Scientific). The RNA integrity of each extract was determined by 16S and 23S rRNA peak examination using a Tape station 2100 (Agilent, Santa Clara, CA).

Since the biofilms were grown on the beef muscle, bacterial RNA may be contaminated with bovine RNA. For this reason, during rRNA depletion, both bacterial and bovine rRNA were removed. The rRNA removal, cDNA synthesis, library preparation and sequencing of the samples was carried out by Next Generation Sequencing facility of Western Sydney University, Australia. The library type was Zymo-Seq Ribofree total RNA. The sequencing was carried out on Illumina HiSeq 2500 sequencer (Illumina, San Diego, CA, USA) and 126 bp length paired-end reads were generated.

### 2.4 Analysis of sequenced reads

The quality of the sequenced reads was assessed using FastQC (Galaxy Version 0.72+galaxy1) function in Galaxy (https://usegalaxy.org.au/). As the sequencing reads are of high quality, without any adaptor contamination and with high per base sequence quality, trimming of the reads was not carried out. The reads were mapped to the *P. fragi* 1793 reference genome (Gene bank accession no NQKS00000000) with the Burrows-Wheeler Aligner (version 0.7.17) using the BWA-MEM algorithm. The mapped reads, which were in BAM format, were visualised using Jbrowse genome browser (version 1.16.4) to determine the efficiency of mapping. The mapping percentages of the total reads to the reference genome ranged between 88 and 96% for the twelve samples (Table S1). Mapped reads were used in subsequent analyses. To estimate the number of genes overlapping with each gene in the reference genome, the SAM/BAM to count matrix of Galaxy was used.

Differential expression was examined using the Voom tool (Galaxy Version 0.28) with a differential count model which provided statistical routines to identify differentially expressed genes (DEGs) between the stages [9]. The gene expression of *P. fragi* in the three stages of the biofilm: (Initiation vs Maturation), (Initiation vs Dispersal), and (Maturation vs Dispersal) were compared pairwise using the DEGUST tool (http://degust.erc.monash.edu/). Genes with a False Discovery Rate (FDR)/adjusted p-value <0.01 and of log_2_-fold change (FC) higher or lower than 2.0 were identified as being significantly differentially expressed. The log_2_-fold change of gene expression shows the fold change in the treatment relative to the control and values in the “Control” are considered as zero. Volcano plots and MA plots were created with Graphpad Prism software (version 7 for Windows, La Jolla California USA) to compare each stage of the biofilm cycle.

### 2.5 Clusters of orthologous groups (COG) function categorization

The DEGs of each stage were categorized in to (COGs) and their correlations with the phenotypic changes in the biofilm cycle were sought. The DGEs of each stage were classified through homology with protein functions determined from clusters of orthologous groups (COG) database. The COG functional categories for the DEGs were obtained by annotating the protein sequences of the reference *P. fragi* 1793 genome using the Eggnog mapper v4.5.1 (http://eggnogdb.embl.de) and genes were placed into COG functional categories. The output of Eggnog provided GO terms, KEGG KO and seed orthologs of the genes. The gene locus tags, log_2_-FC (fold change), FDR, COG categories and gene product description of the highest fifteen differentially expressed genes are provided in Table 1, Table 2, Table 3, Table 4.

**Table 1:** Fifteen upregulated genes with the highest fold changes between biofilm initiation vs maturation.

Locus	Query	FC	FDR	Avg expression	COG cat	Description
CJU75_06635	PAA38363.1	6.27	1.48E-05	8.66	E	catalyzes the transfer of a methyl group
CJU75_22210	PAA32247.1	5.92	0.0078	7.31	S	flp/fap pilin component
CJU75_14375	PAA34791.1	5.58	0.00098	8.00	NA	unknown
CJU75_22215	PAA32248.1	4.87	0.00503	4.07	K	histidine kinase, response regulator
CJU75_22455	PAA32141.1	4.86	0.00488	8.62	J	ribosome modulation factor
CJU75_18560	PAA33363.1	4.78	0.00233	4.92	S	membrane protein
CJU75_05405	PAA38134.1	4.77	0.00164	7.76	E	Glutamate decarboxylase
CJU75_05400	PAA38133.1	4.76	0.00128	6.82	E	Belongs to the glutaminase family
CJU75_06940	PAA37735.1	4.59	0.01133	8.24	E	creatinase
CJU75_15120	PAA34487.1	4.48	0.00063	5.55	P	haloacid dehalogenase-like hydrolase
CJU75_16620 CJU75_19875	PAA33979.1 PAA32825.1	4.42 4.34	0.00015 0.03145	6.68 4.77	C S	arylsulfatase A and related enzymes cytochrome c oxidase accessory protein
CJU75_16745 CJU75_21575	PAA34001.1 PAA32308.1	4.33 4.32	0.01393 0.00014	5.02 3.71	E S	enzyme of the cupin superfamily formation of L-homocysteine from OSHS
CJU75_15085	PAA34481.1	4.30	0.00143	4.38	S	protein conserved in bacteria

**Table 2:** Fifteen downregulated genes with the highest fold changes between biofilm initiation vs maturation.

Locus	Query	FC	FDR	Avg expression	COG cat	Description
CJU75_06450	PAA38329.1	−3.93	0.00435	8.67	S	transmembrane transporter activity
CJU75_21035 CJU75_06445 CJU75_09520	PAA32410.1 PAA38328.1 PAA36861.1	−3.83− 3.84− 3.34	0.00028 0.01031 0.00267	3.30 11.27 9.44	C S P	catalyzes the conversion of L-lactate to pyruvate protein of unknown function ton B dependent siderephore receptor
CJU75_13340	PAA35669.1	−2.97	0.00018	9.44	C	functions as a proton pump across the membrane
CJU75_13335	PAA35668.1	−2.95	3.5E-05	6.44	C	NADP transhydrogenase
CJU75_13330	PAA35667.1	−2.89	0.00011	8.26	C	functions as a proton pump across membrane
CJU75_14700	PAA34854.1	−2.68	0.00485	6.23	IQ	acyl-CoA synthetases AMP-acid ligases II
CJU75_13125	PAA35626.1	−2.66	0.03122	10.08	EI	biotin carboxylase
CJU75_12490	PAA35503.1	−2.59	0.00920	7.29	E	Choline dehydrogenase and related flavoproteins
CJU75_12960 CJU75_08405	PAA35593.1 PAA37329.1	−2.57− 2.50	0.00555 0.00052	8.32 4.94	K S	transcriptional regulator Cupin 2, conserved barrel domain protein
CJU75_12495 CJU75_22975 CJU75_13130	PAA35674.1 PAA31039.1 PAA35627.1	−2.50− 2.48− 2.40	0.01113 0.02555 0.01463	6.69 7.62 9.85	S D CI	gluconate 2-dehydrogenase protein conserved in bacteria AAA domain

**Table 3:** Fifteen upregulated genes with the highest fold changes between biofilm initiation vs dispersal.

Locus	Query	FC	FDR	Avg expression	COG cat	Description
CJU75_22210 CJU75_22455 CJU75_18560 CJU75_16745 CJU75_19875 CJU75_01795 CJU75_08955 CJU75_08945 CJU75_22215 CJU75_06940 CJU75_05405 CJU75_10450 CJU75_10445 CJU75_08960 CJU75_09225 CJU75_05400	PAA32247.1 PAA32141.1 PAA33363.1 PAA34001.1 PAA32825.1 PAA40425.1 PAA37432.1 PAA37430.1 PAA32248.1 PAA37735.1 PAA38134.1 PAA36312.1 PAA36311.1 PAA37433.1 PAA36805.1 PAA38133.1	8.32 8.17 7.29 7.12 7.00 6.95 6.84 6.75 6.68 6.57 6.50 6.47 6.47 6.45 6.43 6.24	5.67E-05 5.24E-07 2.15E-07 1.15E-06 1.8E-06 0.00053 1.97E-06 1.72E-06 7.06E-06 3.19E-05 1.62E-06 2.43E-05 1.63E-05 1.44E-06 3.18E-05 1.43E-06	8.49 10.27 6.14 6.40 6.09 2.75 5.38 6.22 4.97 9.20 8.61 7.39 8.26 7.02 6.23 7.69	S J S E S S I K K E E E E EG C E	flp/fap pilin component converts 70S ribosomes to an inactive dimeric form hypothetical protein in membrane methionine gamma-lyase enzyme of the cupin superfamily catalyzes the degradation of glycine related beta-hydroxyacid dehydrogenases nucleoside-diphosphate-sugar epimerases histidine kinase creatinase belongs to the group II decarboxylase family sarcosine oxidase, subunit beta catalyzes interconversion of serine and glycine belongs to the IlvD Edd family destroys radicals produced within the cells belongs to the glutaminase family

**Table 4:** Fifteen downregulated genes with the highest fold changes between biofilm initiation vs dispersal.

Locus	Query	FC	FDR	Avg expression	COG cat	Description
CJU75_09520 CJU75_06450 CJU75_21035 CJU75_22970 CJU75_06445 CJU75_12665 CJU75_12670 CJU75_12655 CJU75_12960 CJU75_12655 CJU75_12495 CJU75_19920 CJU75_12660 CJU75_14700 CJU75_20405	PAA36861.1 PAA38329.1 PAA32410.1 PAA31038.1 PAA38328.1 PAA35537.1 PAA35538.1 PAA35593.1 PAA35593.1 PAA35535.1 PAA35674.1 PAA32834.1 PAA35536.1 PAA34854.1 PAA32548.1	−4.57− 4.54− 4.13− 3.96− 3.94− 3.94− 3.91− 3.85− 3.86− 3.85− 3.84− 3.84− 3.83− 3.79− 3.75	6.07E-06 2.68E-05 1.49E-06 9.09E-06 0.000138 1.66E-06 2.41E-06 2.21E-06 3.42E-06 2.21E-06 5.32E-06 4.42E-05 2.32E-06 3.82E-07 1.04E-06	3.96 8.38 3.14 6.32 11.21 5.51 6.04 6.00 7.00 6.00 6.00 7.27 5.09 5.66 5.73	P S S S C P P Q K Q S NT P IQ M	tonB dependent siderophore receptor FUSC family, transmembrane transporter activity protein of unknown function (DUF2790) protein conserved in bacteria, ppGpp synthetase, catalyzes the conversion of L-lactate to pyruvate part of the ABC transporter complex in taurine import taurine ABC transporter taurine catabolism dioxygenase transcriptional regulator taurine catabolism dioxygenase gluconate 2-dehydrogenase chemotaxis, protein ABC-type nitrate sulfonate bicarbonate transport system Acyl-CoA synthetases AMP-acid ligases II nucleoside-binding outer membrane

### 2.6 DEG verification using quantitative reverse transcriptase PCR (qRT-PCR)

The RNA samples that were used for sequencing were also used for quantitative reverse transcription PCR (qRT-PCR) to determine if gene expression was consistent between the two approaches. Dilutions were made from the stock RNA to obtain approximately similar amount (800 ​ng) of RNA for reverse transcription. After that, cDNA was synthesized from RNA of three biological replicates using the iScript cDNA Synthesis Kit (Bio-Rad Hercules, CA, cat no 1708890) according to manufacturer’s instructions. Control reactions were performed without reverse transcriptase (RT). Twelve significantly up and down regulated genes (*P* value ​< ​0.01) at each stage of RNA seq reaction were selected for verification by qRT-PCR.

Gene specific primers were designed for selected up and down regulated genes at each stage. The primers were designed using NCBI primer blast and the primers used in this study are listed in Table S2. The 16S rRNA gene was used for normalization within samples. From the cDNA synthesized, qPCR was conducted in a 96 well qPCR plate with three technical replicates for each reaction. No template controls and no RT control were used as control reactions to assess the purity of the reagents and genomic DNA contamination. Real-time PCR was performed using Aria Max (Agilent) real time PCR system under following reaction conditions: 95 ​°C for 3 ​min, 45 cycles consisting of 95 ​°C for 10 ​s and 60 ​°C for 30 ​s, and 72 ​°C for 30 ​s. Melt curve analysis (55–81 ​°C, 0.5 ​°C increments for 30 ​s) was performed to ensure the specificity of primers. The ΔΔCt method was used to analyze the relative expression fold change of the targeted genes [10]. The expression level of each target gene was compared relative to the 16S rRNA internal control gene.

## 3 Results

### 3.1 Biofilm morphology

**Fig. 1:**
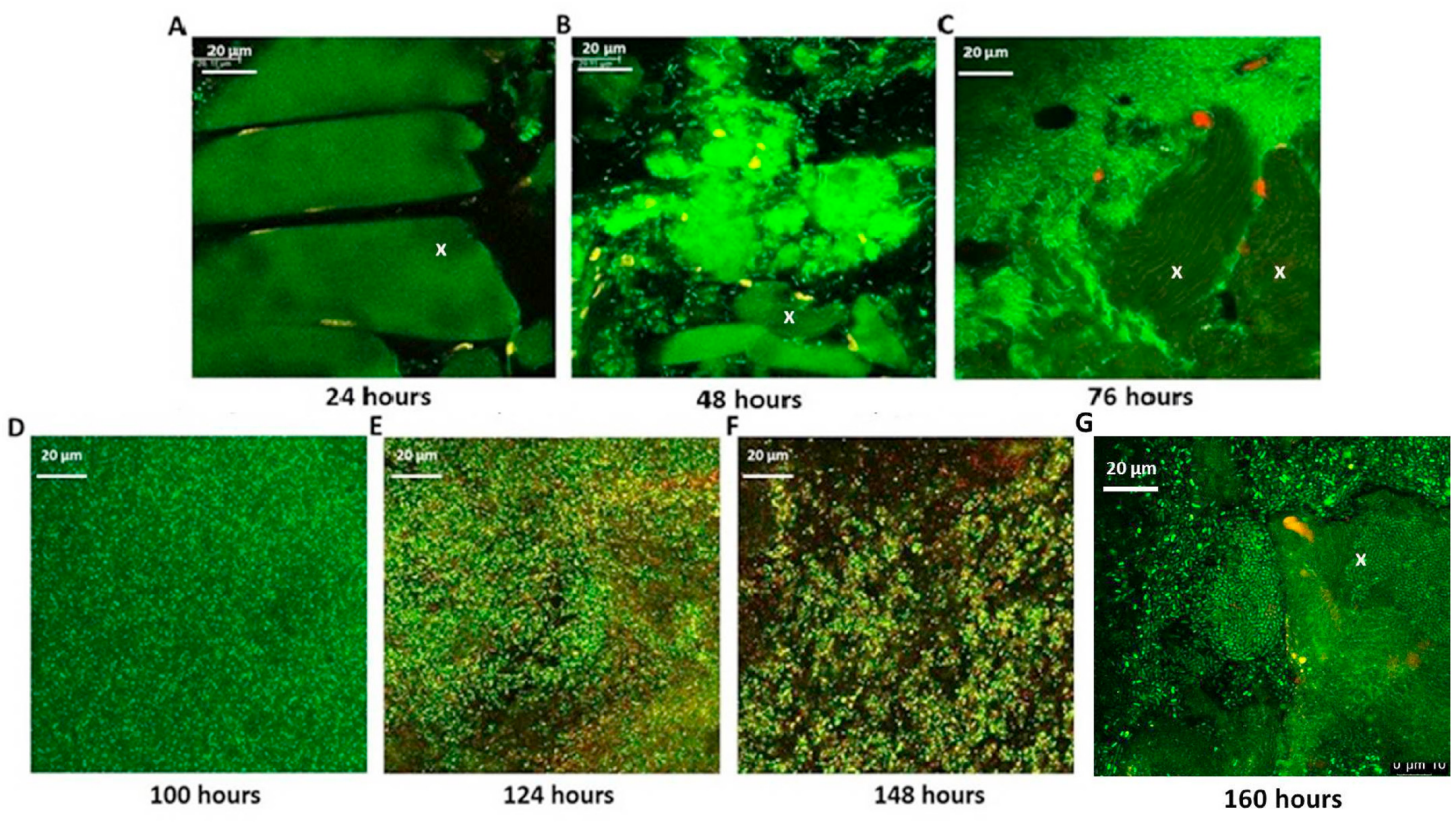
Representative CLSM micrographs of *P. fragi* biofilm cycle on beef incubated at 10 ​°C from 24 ​h to 148 ​h. Small punctuated green staining indicates live cells stained with Syto 9, while dead cells and meat nuclei are stained with PI (red). The Syto 9 stain used to visualize the live cells also stains the meat muscle. The darker green areas marked with a letter X shows the surface of the muscle and the muscle fibres. (For interpretation of the references to colour in this figure legend, the reader is referred to the Web version of this article.)

The representative CLSM images on day 1 (24 ​h), did not show any biofilm growth on the muscle (Fig. 1A). Small flocs of biofilm can be seen on day 2 (48 ​h) and many planktonic bacteria were visible moving between growing biofilms (Fig. 1B). By day 3 (76 ​h), biofilms had started to form covering most of the meat surface and the majority of the cells are alive and can be seen in bright green (Fig. 1C). By this time, the number of CFU in biofilms increased from 10^4^ to ~10^8^ ​CFU ​cm^−2^ and the biofilm biovolume was around 8 ​μm [4]. The maximum population of around 10^11^ ​CFU ​cm^−2^ was reached around day 4 and the biovolume was around 13.5 ​μm. By day 4, the whole muscle surface was covered with the biofilm (100 ​h) (Fig. 1D) The bacterial cells were very tightly arranged with limited inter-cellular spacing. By day 5 (124 ​h), the cells within the biofilm have lost their tight arrangement. Most cells appeared in yellow and dead cells can also be seen. By day 6 (148 ​h), more dead cells were visible in red, and the biofilm structure was breaking apart. (Fig. 1E). By day 7, most cells in the biofilm had been dispersed and the muscle surface was clearly visible again (Fig. 1G). However, part of the biofilm population remained on the meat surface as live cells and the biovolume was around 6.5 ​μm [4].

### 3.2 Comparison of transcriptomic data between the key stages

To find out which genes are significantly up or down regulated and thereby to understand the molecular mechanism behind the biofilm cycle, differential expression analysis was carried out for the sequenced RNA. The extracted RNA from the three stages of the biofilm gave high yields of good quality, non-degraded total RNA with RIN numbers ranging between 9 and 10 (S3). A total of 24–36 million FastQ reads per sample were generated by the high-throughput Illumina sequencing run (S3), (Table S1).

**Fig. 2:**
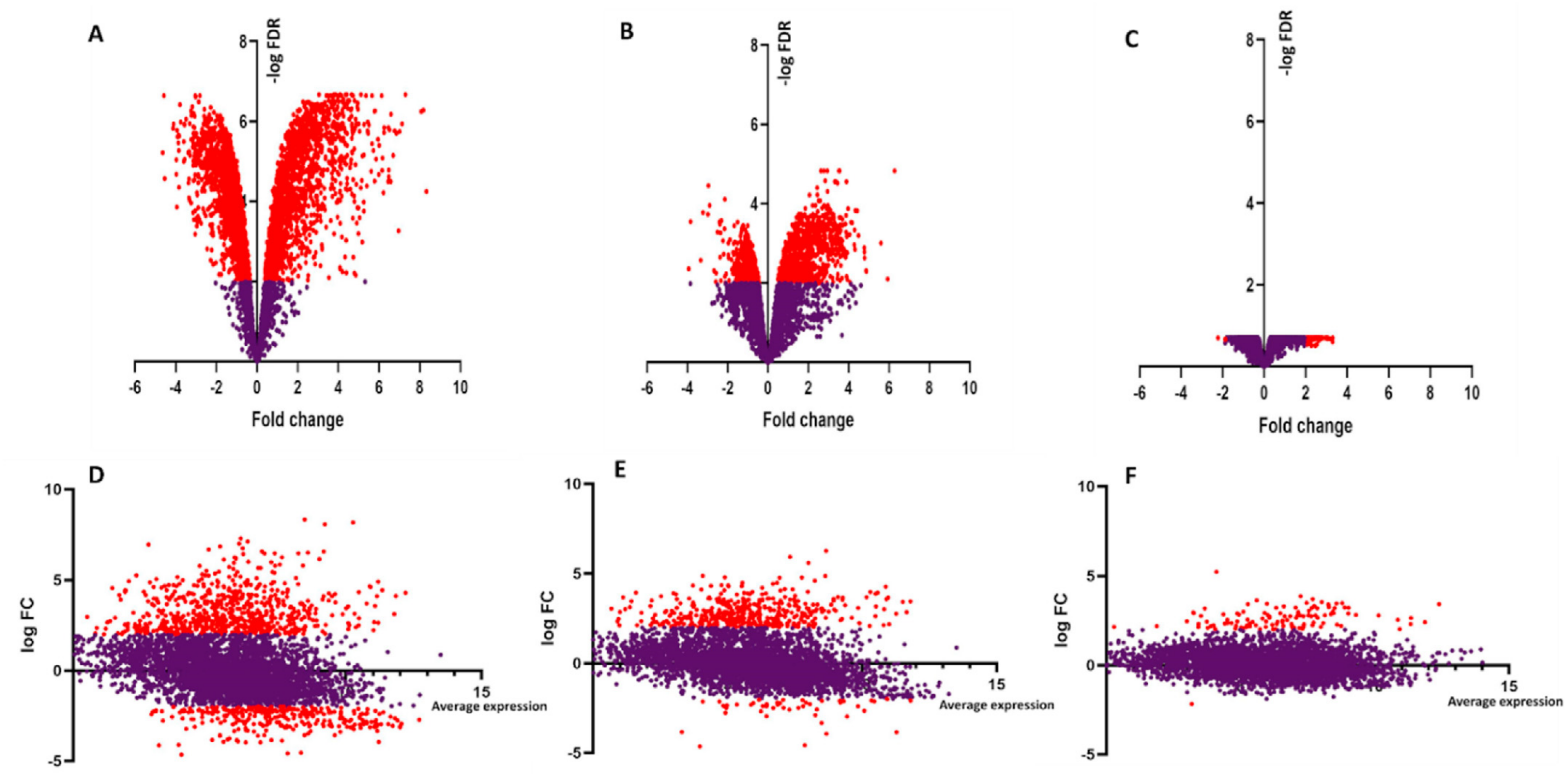
Volcano plots showing differentially expressed genes at biofilm initiation vs dispersal (A), initiation vs maturation (B) and maturation vs dispersal (C). The X axis represents the fold changes and Y axis represents the -log10 FDR. Each dot shows the change in expression in one gene in *P. fragi* genome. Significantly differentially expressed genes (P ​< ​0.01 and of log_2_-fold change higher or lower than 2.0) are highlighted in red and non-significant expressions are presented in purple. MA plots also depict differentially expressed genes at initiation vs dispersal (D), initiation vs maturation (E) and maturation vs dispersal (F). The average expression (over both condition and treatment samples) is represented on the x-axis and Y axis depicts logFC. (For interpretation of the references to colour in this figure legend, the reader is referred to the Web version of this article.)

According to the results depicted by volcano and MA plots, the highest number of differentially expressed genes can be seen between biofilm initiation vs dispersal stages (Fig. 2A and D). The false discovery rate shows the significance of the difference in gene expression between two conditions that were tested at each stage. Compared to other two stages, limited number of differentially expressed genes were present, and many genes were expressed at a lower level between maturation vs dispersal (Fig. 2C and F).

**Fig. 3:**
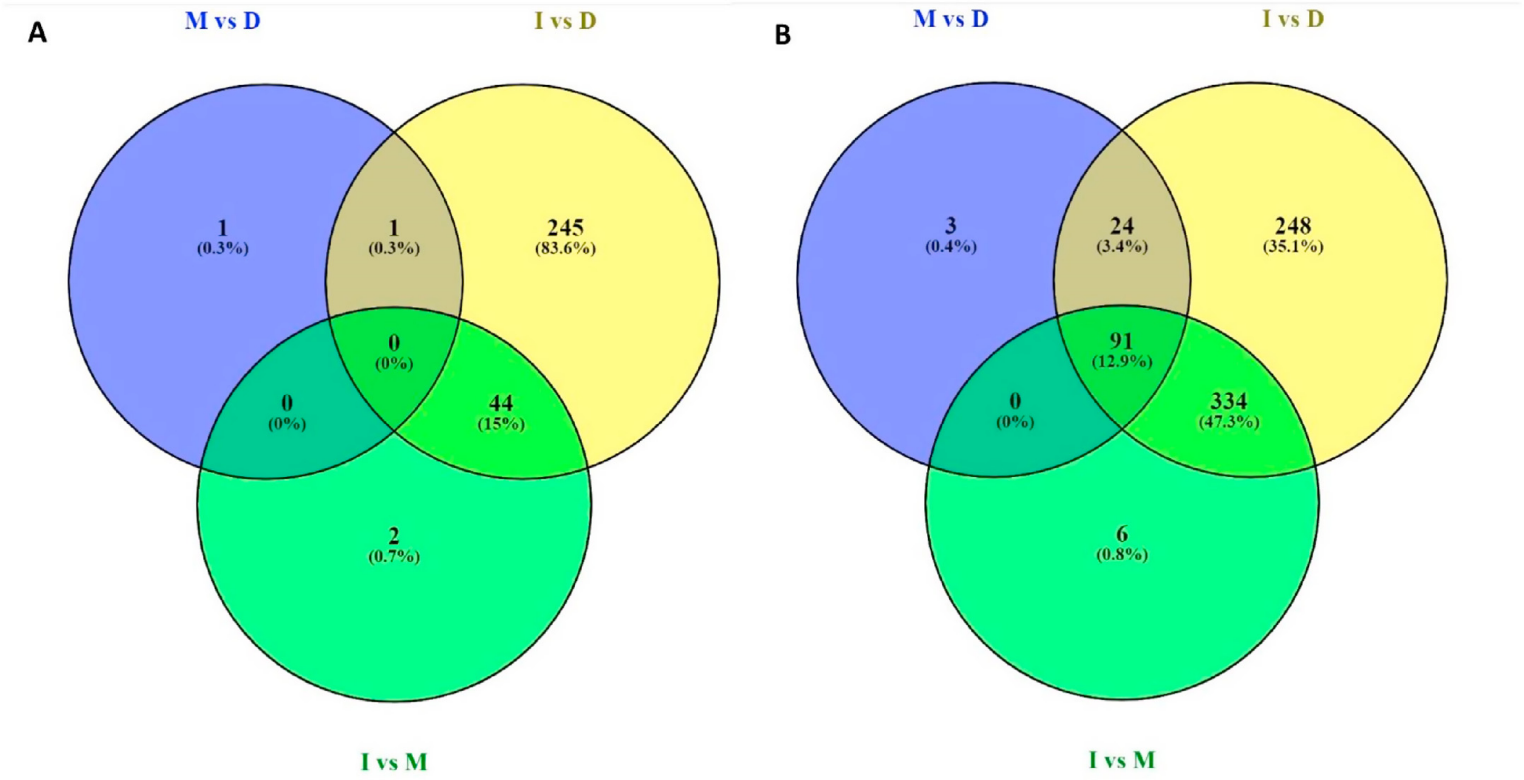
Venn diagram of significantly (P ​< ​0.01) down regulated (A) and up regulated genes (B) at initiation, maturation and dispersal stages of *P. fragi* biofilms formed on chilled beef.

Compared to upregulated genes, downregulated genes had low fold changes in all three stages of the biofilm cycle (Fig. 2). According to the Venn diagram (Fig. 3B), 91 (12.9%) upregulated genes were common to all three main stages of the biofilm whereas no genes were common to all 3 stages in down regulation. Out of total down regulated genes, 83.6% belonged exclusively to initiation vs dispersal stage and 334 common upregulated genes were present between initiation vs maturation and initiation vs dispersal.

### 3.3 Study of the metabolic functions of the DEGs using COG functional categories

The database of Clusters of Orthologous Groups of proteins (COGs) can be used to predict the functions of proteins of bacteria, archaea and eukaryote [11]. In order to get an overview of the increased and decreased metabolic functions at each of the key stages, as well as to understand the molecular mechanisms related to the phenotypic changes that occur in the biofilm, the DEGs were evaluated using the COG database. Also, the two functional categories, COG R and S can reflect the current level of understanding of protein function of *P. fragi* genome [11].

**Fig. 4:**
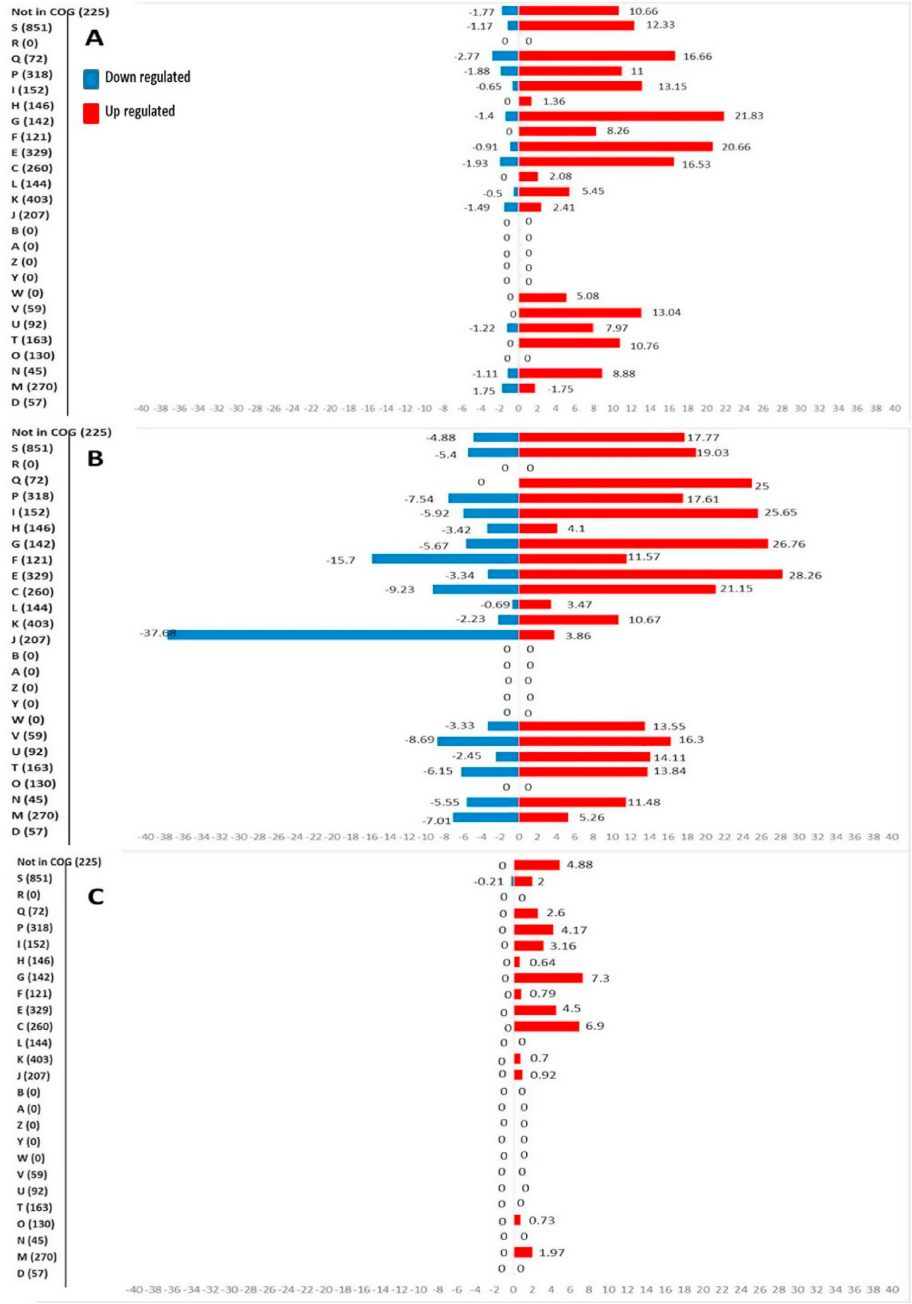
The percentages of significantly upregulated and downregulated genes of *P. fragi* 1793 biofilm formed on chilled beef at initiation vs maturation (A), initiation vs dispersal (B), maturation vs dispersal (C) according to clusters of orthologous groups (COGs).

The DGEs of each stage were classified using the predicted function of the gene products and with protein functions determined from the COG database (Fig. 4, Table S3). The total number of genes in each COG category and the percentages of up and downregulated genes at each stage are listed in Fig. 4. Out of 4333 genes of *P. fragi* 1793 genome, 225 were listed as not in COG where those genes could not be allocated to any COG category.

### 3.4 DEGs in biofilm initiation vs maturation

DEGs in each of the three stages of the biofilm were assessed according to the selected criteria of, (FDR)/adjusted p-value <0.01 and of log_2_-fold change (FC) higher or lower than 2.0. The total number of predicted genes in the *P. fragi* 1793 genome was 4333. A total of 419 genes were significantly differentially expressed between biofilm initiation and maturation which equates to 9.66% of the total genome. Of those 382 genes were significantly up regulated while 37 genes were significantly down regulated. The genes encoding 5-methyltetrahydropteroyltriglutamate-homocysteine S-methyltransferase, flp family type IVb pilin, histidine kinases and ribosome modulation factor were the most upregulated genes with log_2_ fold changes of 6.72, 5.92, 4.87and 4.86 respectively (Table 1). The genes encoding FUSC (fusaric acid resistance) family protein, alpha-hydroxy-acid oxidizing enzyme, tonB-dependent siderophore receptors and heme utilization protein were among the most downregulated with log_2_ fold changes of −3.93, −3.84, −3.34, and −3.21, respectively (Table 2).

Differentially expressed genes between these stages were categorized into 18 COG functional categories. A total of 18 genes had more than one general category letter association in the COG database and were treated as belonging to both. The upregulated DEGS belonged to the following COG categories: carbohydrate transport and metabolism (COG G; 21.83%), amino acid transport and metabolism (COG E; 20.66%), secondary metabolites biosynthesis, transport, and catabolism (COG Q; 16.66%), energy production and conversion (COG C; 16.53%), lipid transport and metabolism (COG I; 13.15%), intracellular trafficking, secretion, and vesicular transport (COG U; 13.04%), function unknown (COG S; 12.33%), inorganic ion transport and metabolism (COG P; 11%) and post-translational modification, protein turnover, and chaperones (COG O; 10.76%) (Fig. 3A). Significantly down regulated genes belonged to the COG categories Q (2.77%), C (1.93%), P (1.88), D (1.77), M (1.75%), J (1.49%), G (1.4%) and T (1.22%), respectively (Fig. 3A). In all the COG categories, the numbers of upregulated genes were higher than downregulated genes.

### 3.5 DEGs in biofilm maturation vs dispersal

According to the selected FDR and log_2_ fold change, 122 genes which equates to 2.81% of the total genome was differentially expressed between maturation vs dispersal. Out of that, 120 genes were up regulated while 2 genes were down regulated. Among them, the ribosome modulation factor, glycine cleavage system protein, heme-binding protein, superoxide dismutase [Mn], were the most upregulated with log_2_ fold changes of 3.30, 3.28, 3.05, 3.03, respectively. At the same time genes related to pyruvate dehydrogenase (PDH) enzyme complex have been upregulated which included, pyruvate 2-oxoglutarate dehydrogenase complex dehydrogenase, dihydrolipoamide acetyltransferase component of pyruvate dehydrogenase complex, pyruvate 2-oxoglutarate dehydrogenase complex and pyruvate 2-oxoglutarate dehydrogenase complex. The fold changes of these genes were 3.45, 3.18, 3.05 and 2.96, respectively. Genes for a hypothetical protein and DUF2292 domain-containing protein were the most downregulated with log_2_ fold changes of −2.67 and −2.17, respectively.

The differentially expressed genes were categorized into 13 functional groups. Between maturation vs dispersal, 6 genes had more than one COG category assigned to them. The highest number of upregulated genes belonged to carbohydrate transport and metabolism (COG G; 7.3%), energy production and conversion (COG C; 6.9%), amino acid transport and metabolism (COG E; 4.5%) and lipid transport and metabolism (COG I; 3.16%). Both the down regulated genes were categorized into COG category S.

### 3.6 DEGs in biofilm initiation vs dispersal

The highest number of differentially expressed genes can be seen between biofilm initiation vs dispersal. According to the selected FDR and fold change, 933 genes which equates to 21.53% of the total genome was differentially expressed. Out of that, 658 were significantly up regulated and 275 genes were significantly down regulated between biofilm initiation and dispersal. Genes encoding Flp family type IVb pilin, ribosome modulation factor, hypothetical protein, methionine gama-lyase, cupin domain containing protein and histidine kinase were the most upregulated with log_2_ fold changes of 8.23, 8.17, 7.29, 7.00 and 6.68, respectively (Table 3). Genes coding TonB-dependent siderophore receptor, FUSC family protein, DUF2292 domain-containing protein, ppGpp synthetase and chromosome partitioning protein ParA were the most downregulated with log_2_ fold changes of −4.57, −4.54, −4.10, −3.969 and −3.698, respectively (Table 4).

Differentially expressed genes between these stages were categorized into 18 functional categories. A total of 25 genes had more than one general category letter association in the COG database and were treated as belonging to both. The majority of upregulated DEGS belonged to the following COG categories: Secondary metabolites biosynthesis, transport, and catabolism (COG Q; 25%), lipid transport and metabolism (COG I; 25.65%), amino acid transport and metabolism (COG E; 20.66%), carbohydrate transport and metabolism (COG G; 26.76%), inorganic ion transport and metabolism (COG P; 17.61%) and energy production and conversion (COG C; 21.15%).

Compared to other two stages, higher numbers of downregulated genes were present between biofilm initiation vs dispersal. At this stage, the highest proportion of down regulated genes belonged to COG J (37.68%) which is related to translation, ribosomal structure and biogenesis. Nucleotide transport and metabolism (COG F; 15.7%), energy production and conversion (COG C; 9.23%) and intracellular trafficking, secretion, and vesicular transport (COG U; 8.69%) were the next highest down regulated categories (Fig. 4B) of this stage.

### 3.7 Verification of DEGs with rt-qPCR

Eleven genes (7 up-regulated and 4 down-regulated) with highest fold changes were selected from three stages of the biofilms cycle and qRT-PCR was performed. The results of qRT-PCR experiments confirmed the gene expression trend observed in RNA seq data (Table 5). Differences in fold changes could likely be due to differences in sensitivity and specificity between RT-qPCR and high throughput sequencing technology.

**Table 5:** Gene expression fold changes generated by RNA seq analysis and qRT-PCR for the selected genes.

Stage of biofilm	Locus tag	Fold change in RNA seq	Fold change in qRT-PCR
Initiation vs maturation	CJU75_22245	4.862458	8.22
	CJU_06635	6.270233	4.02
	CJU75_09520	−3.34048	−1.22
	CJU75_06450	−3.93499	−2.82
Initiation vs dispersal	CJU-22245	8.1725	5.18
	CJU_06635	5.79	9.07
	JU75_19875	7.00	10.22
Maturation vs dispersal	CJU75_03765	−1.8829113	−1.45
	CJU75_13985	−2.2248322	−2.13
	CJU75_17410	3.05179629	5.08
	CJU75_01795	3.28964006	4.89

## 4 Discussion

Biofilm bacteria have different gene expression patterns compared to planktonic bacteria [6]. Even though the duration of each stage can vary based on bacterial species and environmental conditions, all biofilms follow a cycle of bacterial irreversible attachment which initiates biofilm formation, maturation and dispersal of cells to colonise new surfaces [5,8]. Studies have shown that biofilm formation is a genetically regulated process and the gene expression profile at each stage is different [8]. At the same time, transcriptomic analyses of different stages of biofilm development across different bacterial species have failed to show a consistent correlation between all species [6].

Limited information is available about *P. fragi* biofilm formation on actual meat muscle kept under industry applicable conditions. Our main objectives were to study the transcriptomic profile *P. fragi* biofilms in order to identify genes expressions that may aid their survival on meat under low temperature conditions as well as to identify the gene expression during biofilm dispersal stage to come up with mechanisms to limit biofilm and slime formation on chilled meat. Such information can help to design potential compounds with biofilm inhibitory effects.

In this research, RNA extraction time points were selected based on previous research done on *P. fragi* biofilms formed on chilled meat and overlapping of main stages was avoided [4]. According to past research, 48 ​h was selected as the initiation stage as biofilm maturation does not occur during that period and signs of dispersal do not appear at 76 ​h when biofilms are formed at 10 ​°C. Since biofilms were grown on fresh beef muscle, it was important to specifically remove bovine rRNA along with bacterial rRNA. To obtain reliable results, it is important to sequence RNA with high integrity. The RNA isolated in our study was of good integrity and gave high quality reads (S2 & S3). Therefore, post sequence trimming of low-quality reads was not necessary.

### 4.1 DEG in biofilm initiation vs maturation

Biofilms are formed when planktonic bacteria attach to each other or to a surface irreversibly and develop individual microcolonies. Gradually these microcolonies grow as bacteria multiply and form larger structures known as macro colonies. According to our CLSM images, *P. fragi* biofilms start forming small microcolonies around 48 ​h after incubation of meat at chilled/temperature abuse conditions.

In the pairwise comparisons used in this study, biofilm initiation was considered as the control and the gene expression fold changes of the maturation, which was considered as the treatment, was assessed relative to initiation. According to our data, most genes were upregulated as opposed to being down regulated. In addition, the fold changes were higher for upregulated genes than for down regulated genes where the highest downregulation was −0.3.94 and the highest upregulation was 6.27.

Based on the COG categorization of the significantly differentially expressed genes, the majority of upregulated genes at maturation were associated with rapid growth and metabolism of the bacteria. Among them, COGs G and E which represents carbohydrate transport and metabolism, and amino acid transport and metabolism contained the highest numbers of genes. This is most likely due to the rapid increase in the number of bacterial cells within biofilms. At this stage, it is likely that the cells are taking advantage of the nutrient dense environment that they are growing in which contains simple sugars and amino acids. The cells numbers increase from 10^4^ ​CFU ​cm^−2^ to 10^8^ ​CFU ​cm^−2^ within 28 ​h [4]. This requires energy and amino acids for new protein and structural development as well as for exopolysaccharide secretion as the biofilm matures. The genes encoding creatininase enzyme (PAA37735.1) was also significantly upregulated by 6.57- fold. One of the specific characteristics of *P. fragi* is their ability to utilize creatinine and creatine when the substrates are depleted of glucose and lactase, while other psychrotophic spoilage pseudomonads such as *Pseudomonas lundensis* and *P. fluorescence* lack this ability (Drosinos & Board, 1995). When the cell numbers reach around 10^7−8^ ​CFU/cm^2^, meat surface gets depleted of glucose and other simple carbohydrates [12,13]. At the maturation stage, the cell numbers are around 10^7−8^ ​CFU/cm^2^ and creatininase is likely to be used for energy requirement in *P. fragi* biofilms.

Several genes related to taurine metabolism which include taurine transporter ATP-binding subunit, taurine ABC transporter permease, taurine ABC transporter substrate-binding protein and taurine dioxygenase were highly down regulated. To date the effect of taurine on bacterial physiology and biofilm growth has been poorly explored. However, a study on *Acinetobacter oleivorans* have shown that taurine has biofilm inhibitory effects by decreasing total EPS mass and interfering with quorum sensing [14]. Some studies have found that in order for it to be utilized, taurine needs to be transported into the bacterial cells [15]. Taurine is an effective osmoregulatory compound and is used as a compatible solute that can protect proteins, nucleic acids, and membranes from the harmful effects of heat, freezing and drying [16]. Taurine is likely to play a role in protecting the bacteria when they are growing in low temperature conditions. Further studies are necessary to establish the reason for downregulation of taurine transporter systems in *P. fragi* biofilms.

### 4.2 DEGS in biofilm maturation vs dispersal

The lowest percentages of significantly up and down regulated genes can be seen between biofilm maturation vs dispersal which means that these stages are likely to have similar levels of expression. The two significantly down regulated genes belonging to COG S where one gene with −2.67 fold change encoded for a hypothetical protein while the other with −2.17 fold change encoded for a putative DUF 2292 protein, which stands for domain of unknown function.

The highest percentage of upregulated genes belonged to carbohydrate transport and metabolism (COG G) and most of the genes encoded for the major facilitator superfamily (MFS) which facilitates the movement of solutes across the cell membrane in response to chemiosmotic gradients [17]. The second most upregulated COG category was COG C which contained genes that encode hydrolases, oxidoreductases, dehydrogenases. COG C contained genes that act upon radicals such as superoxide dismutase which are normally produced within the cells and are toxic to biological systems. During latter stages of the biofilm, toxic compounds may accumulate, and upregulation of such genes may be necessary.

In the latter stages of the meat grown biofilm, cell numbers reduce after it reaches the population maximum and as observed in CLSM images the biofilm structures begin to break down. When the environmental conditions become unfavourable, a portion of the cells in biofilms disperse and leave the biofilm. For these cells which are enmeshed in a polymeric matrix of polysaccharides, proteins and eDNA to be released, matrix degrading enzymes need to be secreted. The upregulated genes encoded degradative enzymes such as nucleases and hydrolases including aldehyde dehydrogenase, short chain dehydrogenase, aspartate ammonia-lyase, and ureidoglycolate which may aid in degradation of matrix material.

The results further showed that several genes related to pyruvate dehydrogenase (PDH) enzyme complex were significantly upregulated. Past studies have shown that depletion of pyruvate from growth medium impaired biofilm formation in *P. aeruginosa* [18]. Recent studies have shown that the use of exogenous (PDH) enzyme can cause dispersal in existing *P. aeruginosa* biofilms where sessile surface attached bacteria were released in planktonic state [19,20]. The dispersion of biofilms was triggered by PDH through the action of pyruvate depletion. Since several genes related to PDH are significantly upregulated, it can be concluded that the use of exogenous PDH to deplete pyruvate from inside the biofilm may cause dispersal in *P. fragi* biofilms as well.

### 4.3 DEGs in biofilm initiation vs dispersal

. The majority of genes significantly differentially expressed at initiation vs maturation stage are similar to the genes significantly differentially expressed at initiation vs dispersal stage. However, at dispersal stage the genes have a higher fold changes as the biofilm is further matured. In the list of significantly upregulated genes, the highest fold change of 8.49 was observed in genes encoding Flp family type IVb pilin related to locus tag CJU75_22210 (PAA32247.1). Another locus tag, CJU75_08345 (PAA37317.1.) also encoding for Flp family type IVb pilin protein has been significantly upregulated by a fold change of 5.77. Type IV pilins (T4P) are small structural proteins which does various functions in bacteria. Its well-characterized roles include adherence to living and non-living surfaces as well as to other bacteria [21,22]. The adherence to surfaces and to each other are crucial steps in biofilm formation. It has been reported that *P. aeruginosa* mutants lacking T4P are deficient in biofilm formation and biofilm structures are impaired [23,24]. The T4Ps are likely to be key genes that govern *P. fragi* biofilm formation.

The second highest upregulated gene with 8.17-fold change was the ribosome modulation factor (RMF) which is a ribosome-associated protein. Studies on RMF mutants and parent strains have found that survival of *Escherichia coli* under extreme environmental conditions is higher in parent strains [25]. Efficient functioning of the ribosomes is essential for protein synthesis, growth and survival of the bacteria. It has been found that RMF protects the ribosomes and helps to maintain its structure and function which maintains the cell viability under cold environmental conditions Our result suggest it is possible that RMF provides stability for ribosomes when the bacteria are grown under chilled temperature conditions which are stressful to the bacteria.

Genes coding for methionine gamma-lyase, were also highly upregulated with a fold change of 7.12. This enzyme degrades sulfur-containing amino acids to α-keto acids, ammonia, and thiol. Also, genes such as L-serine ammonia-lyase and aspartate ammonia-lyase are also highly upregulated at this stage which catalyze amino acids and release NH_3_ [26,27]. By this stage bacteria in the biofilm have utilized glucose and simple sugars on meat and have begun to degrade proteins via proteolysis. The putrid odors released during the latter stages of spoiled, chilled meat, is due to the catabolism of amino acids from muscle protein.

Among the highly down regulated genes at the biofilm dispersal stage, genes encoding for TonB-dependent siderophore receptor (PAA36861.1) had a fold change of −4.57. TonB dependent siderephore receptors are bacterial outer membrane proteins that bind and transport ferric chelates [28]. Bacteria use siderophores to chelate iron. Due to their molecular weight, siderophores are not able to diffuse through the porins present in the outer membrane of Gram-negative bacteria into the cytoplasm. Bacteria use TonB dependant receptors to actively transport siderophores into the periplasm [8]. In the periplasm siderophores are bound by a periplasmic binding protein and delivered to an inner membrane by ABC transporter. In our study genes encoding the ABC transporter permease (PAA35243.1) were also been significantly down regulated. Although it was previously suggested that *P. fragi* did not produce siderophores [29,30], the results of this study clearly indicate that *P. fragi* contain genes that encode for siderophores. Also, a recent study has found that *P. fragi* produce a vibrioferrin-mediated iron acquisition system under iron starvation conditions [31], but no information was available on siderophore production under biofilm conditions.

Genes that encode FUSC proteins have also been down regulated with −3.52-fold change. Studies have found pathogenic bacteria such as *Pectobacterium* remain virulent under iron limiting environments using iron containing proteins such as ferredoxin, pirated from their hosts [32,33]. This import pathway is facilitated by FUSC proteins and it may play a role in iron acquisition in *P. fragi* as well. Currently no information is available on functions FUSC family proteins in *P. fragi.* Our results further show that heme utilization protein (PAA32411.1) was also significantly down regulated. Certain pathogenic bacteria use heme as an alternative source of iron and when they are incapable of producing siderophores [34].

Based on these results, it is likely that *P. fragi* use several iron uptake systems for its function and its regulation varies as the biofilm matures. Iron is an essential micronutrient required for bacterial growth and studies have found it to be is essential for biofilm formation in *P. aeruginsa* [35]. According to our results, it is clear that as *P. fragi* biofilm matures, it down regulates the genes required for iron uptake. The exact reason for this is currently unknown. However, high concentrations of iron can be toxic to bacteria as it can lead to reactive oxygen species [36]. Also, it has been shown for *P. aeruginosa* that once the biofilms are formed, the matrix exopolysaccharides can store iron [37]. A study on *P. aeruginosa* by Yang, Barken [38] showed that high concentrations of iron supress extra-cellular DNA release in biofilm which is a crucial part of its structure and it weakens the biofilm structural development. When *P. fragi* is growing in an iron rich environment like meat, they may down regulate genes that govern iron acquisition systems in order to control high concentrations of iron in the biofilm matrix.

A recent study has found that the exposure of *P. aeruginosa* biofilms to exogenous nitric oxide (NO) inhibited the expression of iron acquisition-related genes and the production of siderophores [35]. Nitric oxide is a known dispersal agent in *P. aeruginosa* biofilms. Similarly, when *P. fragi* biofilms matures the release of nitrogenous byproducts due to proteolysis of meat also increase and this may trigger down regulation of iron acquisition related genes. Therefore, the use of exogenous NO releasing food grade compounds with a combination of high concentrations of iron may have potential in controlling biofilm formation on meat.

Compared to other stages of the biofilm cycle, the highest number of downregulated COGs can be seen between initiation vs dispersal stage. It is interesting to see that COG J which is related to translation, ribosomal structure and biogenesis contained the highest percentage of genes. Most significantly down regulated genes of COG J coded for ribosomal proteins, ribosome binding proteins, rRNA binding proteins and accessory proteins required for ribosome assembly. It can be hypothysed at the dispersal stage of the biofilm cycle, protein production ceases or gets reduced considerably. Also COGs F and C had the next highest down regulated percentages which are related to nucleotide transport and metabolism and energy production and conversion, respectively. Genes listed in COG F contained genes encoding for catalytic enzymes such as adenylosuccinate lyase subfamily, dGTPase family, Nudix hydrolase family and purine pyrimidine phosphoribosyl transferase family.

A higher percentage of genes categorized in COG D which represents cell cycle control, cell division, chromosome partitioning was down regulated than up regulated. It indicates that once the biofilms reach their population maximum of around 10^11^-10^12^ ​CFU^−2^, the cell division and protein synthesis get ceased. The CLSM images corelates well with the RNA seq results. At around 124 ​h, there are less green coloured (live) cells in the degrading biofilm. Most cells in the biofilm have taken yellow and red colours which indicates the cells are losing their viability and dying.

The 91 common upregulated genes between three stages of biofilm cycle (Fig. 4) may be responsible for basic metabolic functions. Also, there were 225 genes that could not be classified to any of the current COG functional categories. Aside from the genes which have known functions, there were many hypothetical proteins significantly differentially expressed in all three stages of the biofilm indicating that they are likely important in biofilm formation. However, their exact functions remain unknown.

## 5 Conclusion

This study has used an experimental model that closely mimics practical industry conditions, and the data provides a molecular basis for *P. fragi* biofilm formation on chilled meat. Our study helped to identify the key genes that are up and down regulated at important stages of a biofilm formed on beef muscle under chilled conditions. This study investigated a global gene expression for a population of bacteria within the biofilm. However, within a dense biofilm gene expressions can sometimes vary between upper and bottom layers. Single cell RNA sequencing could be a solution for this issue but can be quite complicated when the biofilms are formed in complex meat tissue. Biofilms are formed when planktonic bacteria attach irreversibly to a surface. Planktonic bacteria were not included in this project due to excessive variability as planktonic bacteria are grown in broth media which is an environment considerably different to complex meat muscle. The composition of the nutrients and its availability to bacteria in a liquid broth is much different to those on a solid muscle surface and there will be too many variables. Thus the comparison would not be useful.

The similarity in genes expressed between maturation and dispersal is most likely due to the selected of time points. More differences could have been detected if RNA were extracted from a very mature, degrading biofilm. However, it is not practical to extract RNA at late dispersal stage as majority of cells have lost its viability and rest are dead. RNA extraction from dead cells was avoided by selecting 115 ​h for extraction.

Knowledge about the gene expression can help explain many of phenotypic changes that occur during biofilm formation on meat. Understanding the cues that can trigger gene expression related to biofilm dispersal may aid in developing effective control measures. The ribosome modulation factor and creatininase aided the survival of *P. fragi* under low-temperature conditions. We have also found that iron uptake systems get significantly down regulated as the biofilm matures. High concentrations of iron and exogenous NO can be specific targets that may be useful in controlling *P. fragi* biofilms. The use of pyruvate dehydrogenase could also be a promising approach in removal of *P. fragi* biofilms via triggering dispersal.

## Credit author statement

Conceptualization and Methodology: Nirmani Wickramasinghe, Scott Chandry, Gary Dykes, Funding acquisition: Gary Dykes, Data curation and analysis:, Nirmani Wickramasinghe, Scott Chandry, Writing and original draft preparation: Nirmani Wickramasinghe, Supervision.: Scott Chandry, Writing- Reviewing and Editing : Scott Chandry, Gary Dykes, Joshua Ravensdale, Rani Cooray.

## Funding

This research was funded by the Australian meat processors corporation (AMPC).

## Data Availability Statement

The sequencing data is available to the public and can be accessed via https://www.ncbi.nlm.nih.gov/geo/info/update.html.

## Declaration of competing interest

The authors declare that they have no known competing financial interests or personal relationships that could have appeared to influence the work reported in this paper.
