# Supplementary material for: Transcriptional profiling of biofilms formed on chilled beef by psychrotrophic meat spoilage bacterium, Pseudomonas fragi 1793
Source: Biofilm. 2021 Feb 17;3:100045. doi: 10.1016/j.bioflm.2021.100045 (PMC7921472; doi:10.1016/j.bioflm.2021.100045)
Supplement: Multimedia component 1 [file mmc1.docx]

| **SUPPLEMENTARY MATERIAL 1**  **Table S1.** Primers used for selected genes in rt-QPCR   \| **Locus tag** \| **Forward primer** \| **Reverse primer** \| \| --- \| --- \| --- \| \| CJU75_19875 \| CCTCTGGGGTGATGATGCAATA \| ACTCGAAGGCGACCCCCATA \| \| CJU75_22245 \| GCGTTCCTTCGCGGATATCA \| TCGTTGAGTCTGTGGATGCC \| \| CJU75_06635 \| TCTGGAACAGGCAGGGATCA \| TTTCATCCCCCACACCCGAG \| \| CJU75_06635 \| TCTGGAACAGGCAGGGATCA \| TTTCATCCCCCACACCCGAG \| \| CJU75_22245 \| CCTGACTGAAGAAACCCGCA \| CGCTCTTCGTCTTCGCTGAT \| \| CJU75_06450 \| TATAGCACTGTCGGCCCTCT \| GCATAAGCAAATCCCTGGGC \| \| CJU75_09520 \| GTCCGAATTGGTTGAGAGCGT \| TGCCAGTTTGAGTTTCAGCGT \| \| CJU75_01795 \| GGGTGATGTGGTGTTTGTGC \| ATGGGTGAGGCATTGACCAG \| \| CJU75_17410 \| ATGCAACTCAGGGTGCCAAT \| CCAACTGCACGTTATCCAGG \| \| CJU75_13985 \| AGGACGAAGCAACCATCCTG \| GGGTTTTGCAGGCGGAATTT \| \| CJU75_03765 \| GTGCCATAGAAAACCATGTCCG \| CAAAGGTGTTTTCGGAGTCGC \| \| 16S rRNA gene \| GTCACCGGCAGTCTCCTTAG \| ATTGGTGCCTTCGGGAACAT \| |
| --- | --- | --- | --- | --- | --- | --- | --- | --- | --- | --- | --- | --- | --- | --- | --- | --- | --- | --- | --- | --- | --- | --- | --- | --- | --- | --- | --- | --- | --- | --- | --- | --- | --- | --- | --- | --- | --- | --- | --- |

**Supplementary material 2**

**Quality reports of extracted total RNA from biofilms formed on chilled beef**

**Supplementary material 3**

**Illumina HiSeq 2500 sequencing results**

**Supplementary material 4**


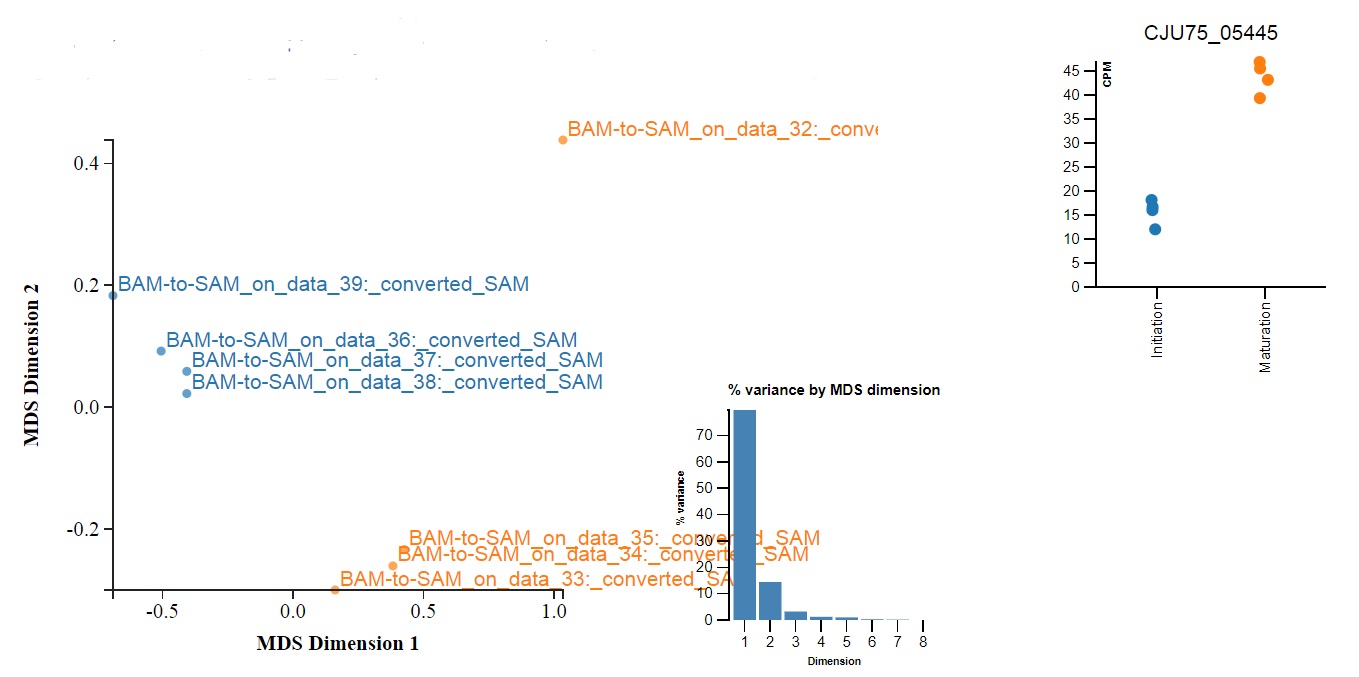


**Fig S1.** Multi-dimensional scaling (MDS) plots of BAM-to SAM converted data of the four biological replicates of biofilm initiation and maturation


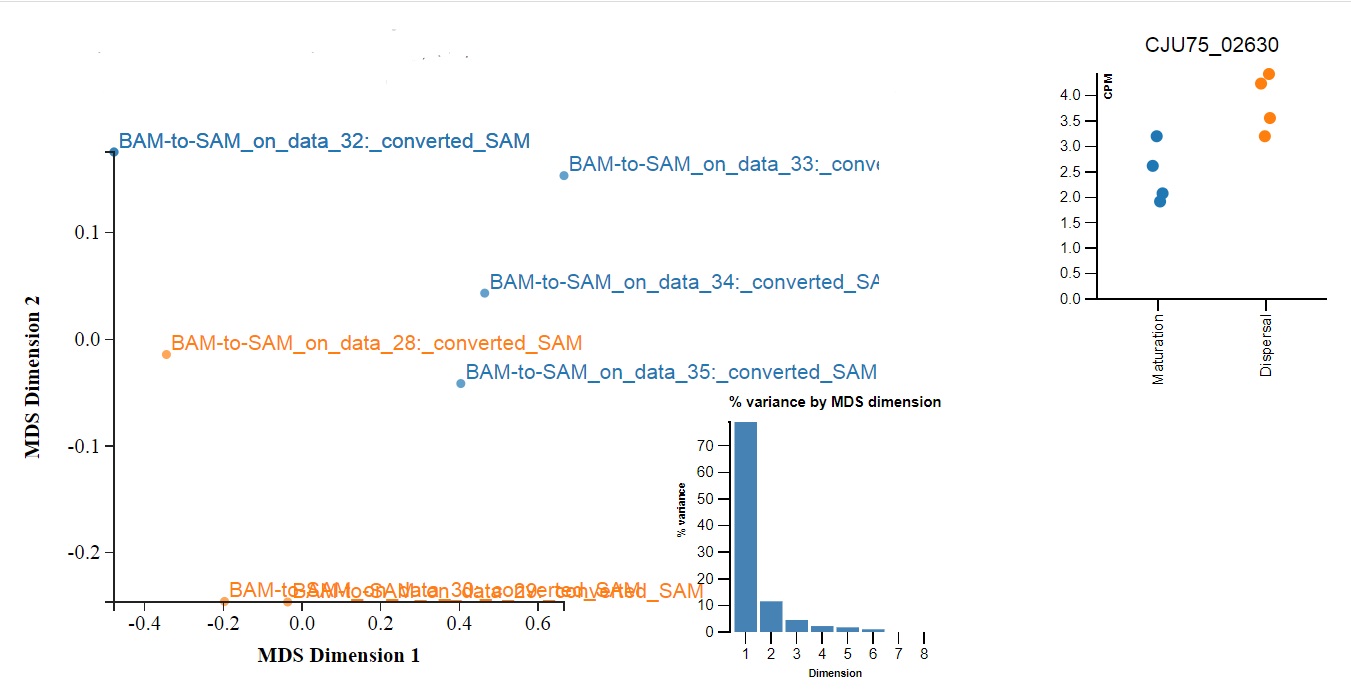


**Fig S2.** Multi-dimensional scaling (MDS) plots of BAM-to SAM converted data of the four biological replicates of biofilm maturation and dispersal


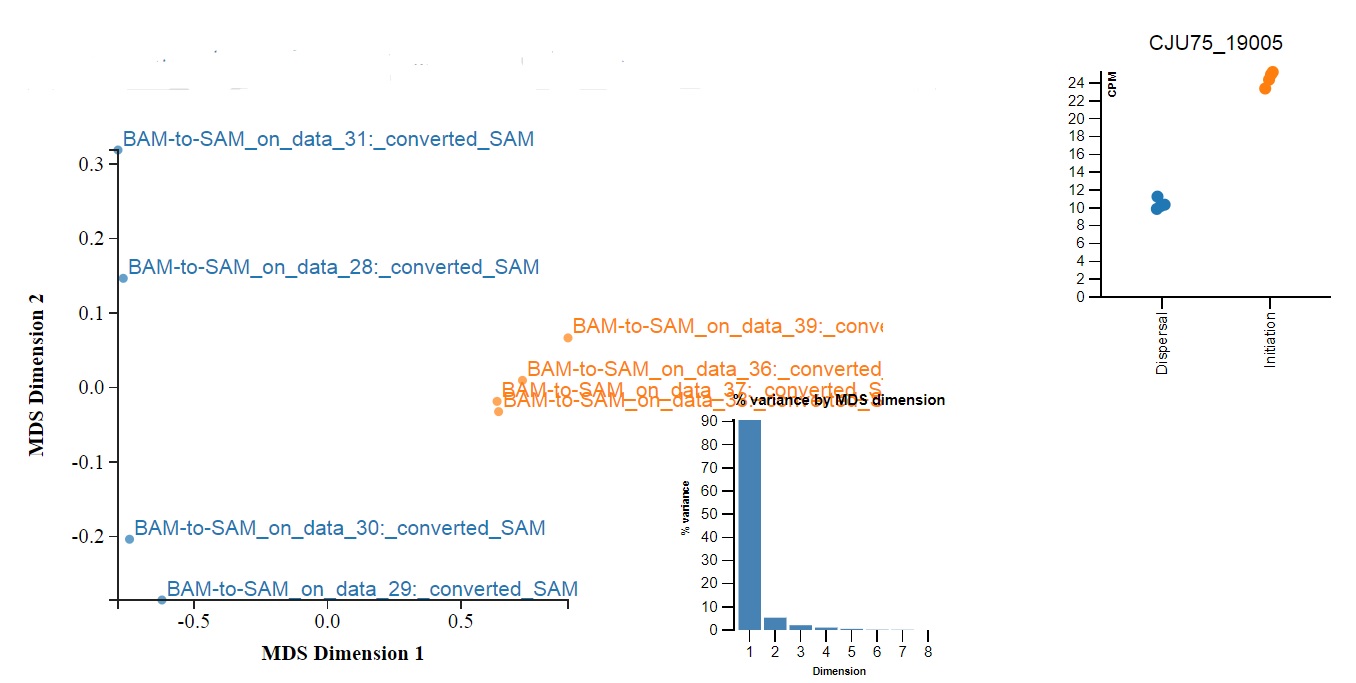


**Fig S3**. Multi-dimensional scaling (MDS) plots of BAM-to SAM converted data of the four biological replicates of biofilm dispersal and initiation
